# Retraction Note: The suppressing role of miR-622 in renal cell carcinoma progression by down-regulation of CCL18/MAPK signal pathway

**DOI:** 10.1186/s13578-023-00976-x

**Published:** 2023-02-09

**Authors:** Tian Li, Xiangzhou Sun, Kewei Xu

**Affiliations:** 1 Department of Urology, The Fifth Affiliated Hospital of Guangzhou Medical University, 621 Gangwan RD, Huangpu District, Guangzhou, 510700 Guangdong China; 2 Minimally Invasive Technique and Product Translational Center, Guangzhou Medical University, Guangzhou, Guangdong China; 3 Department of Urology, The First Affiliated Hospital of Sun Yat-Sen University, Guangzhou, Guangdong China; 4 Department of Urology, The Second Affiliated Hospital of Sun Yat-Sen University, Guangzhou, Guangdong China

**Retraction to: Cell Biosci (2018) 8:17** **https://doi.org/10.1186/s13578-018-0212-8**

The Editor-in-Chief has retracted this paper. After publication, concerns were raised around the similarity between the rightmost ACHN panel of Fig. 4f and the TGF-B1 + Celecoxib panel in Fig. 5B in a previously-published article by different authors [1]. The Editor-in-Chief therefore no longer has confidence in the results and conclusions of this article. The authors have not responded to any correspondence from the editor about this retraction.
